# Relationship of the Lumbar Lordosis Angle to the Level of Termination of the Conus Medullaris and Thecal Sac

**DOI:** 10.1155/2014/351769

**Published:** 2014-07-03

**Authors:** C. D. Moussallem, H. El Masri, C. El-Yahchouchi, F. Abou Fakher, A. Ibrahim

**Affiliations:** ^1^Department of Orthopedic and Spine Surgery, Center of Physical Medicine and Rehabilitation, Ain Wazein University Hospital, Ain Wazein, Lebanon; ^2^Department of Surgery, Lebanese University, Beirut, Lebanon; ^3^Department of Anesthesia, American University of Beirut, Beirut, Lebanon; ^4^Department of Radiology, Ain Wazein University Hospital, Ain Wazein, Lebanon; ^5^Department of Orthopedic Surgery, Lebanese University, Beirut, Lebanon

## Abstract

The level of termination of the conus medullaris (CM) and thecal sac (TS) is subject to variations. We try to correlate in this study these variations with the lumbar lordosis angle (LLA) using MRI scans. A retrospective study was conducted using available MRI scans of the lumbar spine. The CM level of termination (CMLT) and the TS level of termination (TSLT) were identified according to a vertebral level after dividing it into 3 parts. The LLA was also identified for each individual. Linear regression models were fitted to the data available on 141 individuals. Of these 70 were males and 71 were females. The most common site of CMLT was at the upper third of L1 (32.6%) and that of the TSLT was at the middle third of S2 (29.8%). The mean LLA was 46° (20°–81°). The most proximal CMLT was at the upper third of T12, whereas the most distal one was at the upper third of L2. The most proximal TSLT was at the upper third of S1, whereas the most distal one was at S3-S4 disc space. The CMLT showed a positive correlation with the LLA. In conclusion the CMLT and TSLT may be related to variations of the LLA.

## 1. Introduction

Anatomical planes used in clinical practice and spinal anatomy teaching are largely derived from cadaveric studies [1]. Numerous variations exist in the position of the conus medullaris with a peak incidence at the lower third of L1 but can range between the middle third of T12 and the upper third of L3 [2, 3]. Similar disparities are also described concerning the TSLT which has been described in standard textbooks and cadaveric studies at S2 [4, 5]. However, it may extend caudally beyond the S2 level [6, 7].

On the other hand, variations of the LLA also exist and are defined as ranging from 30° to 75° in normal individuals [8]. The question we ask in this paper is whether these variations are related. Our work tries to find the relationship between the CMLT, TSLT, and LLA based on magnetic resonance imaging in normal living individuals. These findings may help us to better understand the anatomy of the spine and not to rely only on cadaveric studies.

## 2. Materials and Methods

After approval by our institutional research and ethic committee, a retrospective study was conducted using lumbar magnetic resonance imaging from our radiology department data available from September 2012 to February 2013. Patients having spinal anomalies such as fractures or deformities were excluded from our study as well as patients with previous spinal surgeries, intra- or extradural tumors, spinal degenerative changes, or obvious anatomical abnormalities. Individuals with lumbosacral transitional segment anomalies were also excluded. All patients aged less than 18 years were also eliminated since we considered that spinal maturity is not complete in this age group.

**Figure 1:**
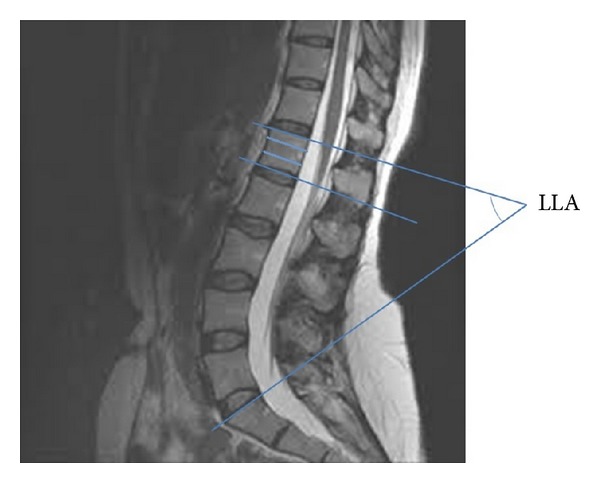
T2 weighted MRI of the lumbar spine showing the method of determination of the CMLT in relation to a vertebral segment.

Multiplanar reconstruction on T1 and T2 weighted MRI images were performed using a 3 Tesla machine. The CMLT and TSLT were identified on sagittal images after dividing each vertebral level (vertebral body with its corresponding disc space) into four parts: upper third, middle third, lower third, and the intervertebral disc space (Figure 1). The CMLT and TSLT were defined as the most distal part of the spinal cord and dural sac that can be visualized on sagittal imaging. A similar method was done previously by Saifuddin et al. [2] and Kim et al. [9]. The LLA was measured on a sagittal scout view going from the superior endplate of L1 to the superior endplate of S1. It is worthy to mention that no cases of lumbar sacralization or sacral lumbarization or any transitional anomalies were identified that may influence the counting of the vertebras (these were ruled out using the initial scout view that serves for counting purposes). Any patient with fat in the filum terminale that could represent a tethered cord or a thickened filum with more than 2 mm in diameter was also excluded. The data was analyzed using Pearson's linear correlation coefficients. Probability values (*P* ≤ 0.05) were considered statistically significant.

## 3. Results

**Figure 2:**
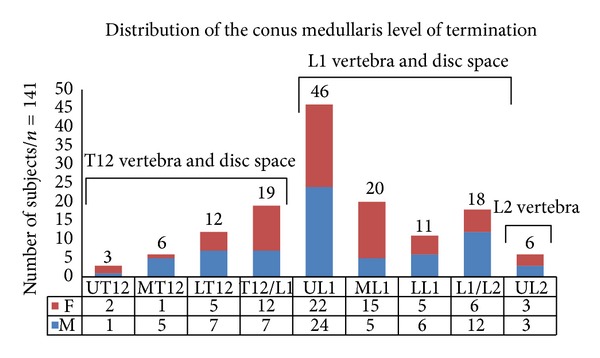
Distribution of the conus medullaris level of termination.

**Figure 3:**
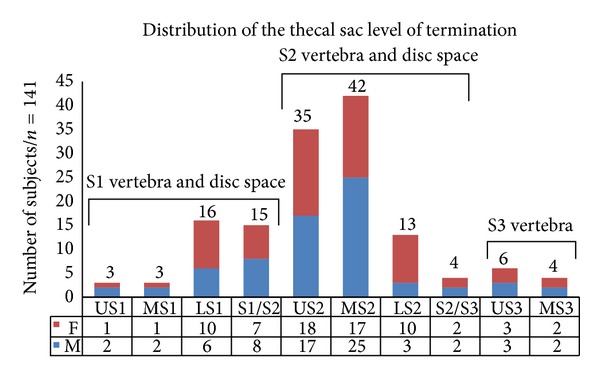
Distribution of the thecal sac level of termination.

193 scans were available for initial review and after applying the exclusion criteria, the final group consisted of 141 individuals: 70 were males (49.65%) and 71 were females (50.35%). The most common site of termination of CM was at the upper third of L1 (32.6%) and that of TS was at the middle third of S2 (29.8%). The most proximal location of the CMLT was at the upper third of T12, whereas the most distal location was at the upper third of L2. The most proximal location of the TS was at the upper third of S1, whereas the most distal one was at S3-S4 disc space (Figures 2 and 3). The mean LLA was 46° (20°–81°).

**Figure 4:**
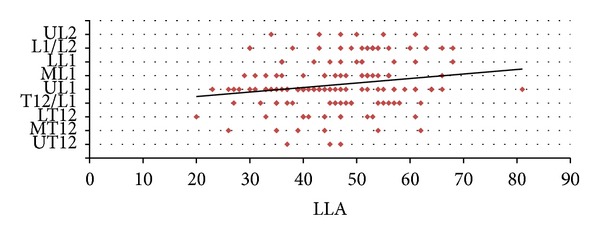
Correlation between the conus medullaris level of termination and the thecal sac level of termination.

**Figure 5:**
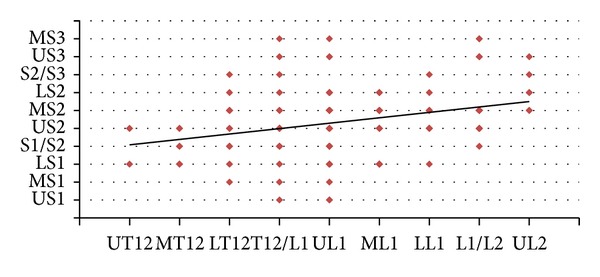
Correlation between the lumbar lordosis angle and the conus medullaris level of termination.

**Figure 6:**
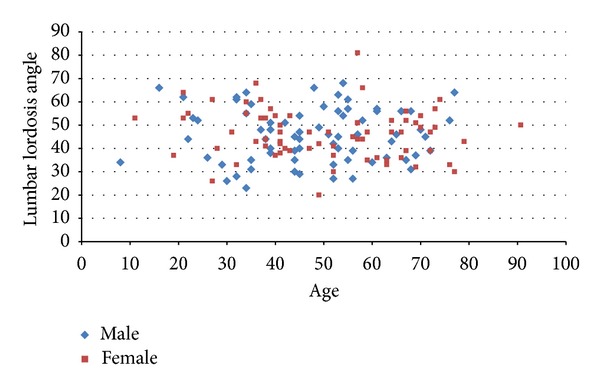
Relationship of the lumbar lordosis angle to age for male and female individuals.

The Pearson product-moment correlation coefficient, a measure of the linear dependence between two variables, was measured between the CMLT and TSLT and showed a positive significant medium correlation (*r* ^2^ = 0.32; *P* = 0.0012) (Figure 4). The LLA showed a positive significant medium correlation with the CMLT (*r* ^2^ = 0.48; *P* = 0.0014) (Figure 5). On the other hand, no correlation was found between the LLA and the TSLT. In addition, we could not find also any difference of the CMLT or the TSLT in relation to age or sex. The LLA in relation to age and sex is shown in Figure 6.

## 4. Discussion

Large variations exist in the literature concerning the position of the conus medullaris and the thecal sac [9, 10]. The first available data concerning the location of the CM was done by Thomson after studying 198 adult cadavers in 1893 [11]. Since that time, most of our anatomy teaching was based on cadaveric studies with wide unexplained variations in the CMLT and TSLT ranging from T12 to L3 [9] and L5 to S3 [12], respectively.

In our study, we found that the most common site of CMLT was at the upper third of L1 in 32.6% of the cases and that of TS was at the middle third of S2 in 29.8% of the cases. If we take into account each vertebral segment (a vertebra and its corresponding disc) as a single anatomical entity, the most common CMLT will be at the L1 segment in 67.37% (28.346% at T12; 4.27% at L2) of the cases and that of the TSLT will be at the S1 segment in two-thirds of the cases, 66.7% (26.24% at S1; 7.06%). Grossly, most of our data were in accordance with the literature.

This is the first anatomical study that tries to correlate the CMLT and the TSLT with the LLA. Since the LLA has a wide range of variations in the adult population and can change from one individual to another, we postulated that these variations may affect the variations of the CMLT and TSLT. In fact, the LLA has a variable range from 30° to 75° in the normal adult population [8]. To the best of our knowledge, no one of the previous studies concerning this topic evaluated the effect of the LLA on the CMLT and TSLT even though these levels are referenced to their corresponding vertebral segment. We demonstrated in our work that the CMLT tends to move more distally with an increasing LLA and vice versa. We did not find any correlation between the LLA and the TSLT which could be explained by the fact that the thecal sac is located within the sacral canal and not the lumbar spine.

In addition, there was a positive significant medium correlation (*r* ^2^ = 0.32; *P* = 0.0012) between CMLT and the TSLT. A similar robust correlation was also observed between the CMLT and TSLT (*r* ^2^ = 0.309; *P* = 0.001) in a study done by Soleiman et al. [13]. The distance between the CMLT and TSLT of the same patient was never less than 5 vertebral segments or more than 7 segments. The mean distance between the TSLT and the CMLT was of 6 vertebral segments. We did not find in this study any correlation between age, sex, and CMLT or TSLT. Nevertheless, previous studies showed that CMLT was lower in female patients but this did not affect the TSLT and that age may influence the level of TSLT [9, 13]. Similar to this work, a recent study done by Moussallem et al. [14], published in* Clinical Anatomy*, revealed that the variations of the LLA closely correlate with the position of the abdominal aortic bifurcation and inferior vena cava confluence level. As for the limitation of this study, all MRI scans were performed in a supine position which may alter the LLA and the data was gathered in a retrospective manner. In addition, this could have many potential clinical implications specially while providing spinal anesthesia and in different aspects of spine surgery.

## 5. Conclusion

This study showed that the variations of the level of termination of the CM may be related to variations of the LLA. When the LLA tends to increase, the level of termination of the CM tends to be located more distally and vice versa. The CMLT and TSLT are also related. In conclusion, the wide variations of the level of termination of the CM may be explained by the variations of the LLA from an individual to another.
